# Supplementary material for: Green Synthesis of Silver Oxide Nanoparticles from Mauritia flexuosa Fruit Extract: Characterization and Bioactivity Assessment
Source: Nanomaterials (Basel). 2024 Nov 22;14(23):1875. doi: 10.3390/nano14231875 (PMC11643947; doi:10.3390/nano14231875)
Supplement: Supplementary file 1 [file nanomaterials-14-01875-s001.zip › nanomaterials-3277367-supplementary.pdf]

SUPPLEMENTARY MATERIAL

Table S1. Percentage of Biofilm Inhibition Activity of Mf-Ag_2ONPs

**Mf-Ag_2ONPs**	***Staphylococcus aureus*** **ATCC 25923**		***Listeria monocytogenes*** **ATCC 13932**		***Pseudomonas aeruginosa*** **ATCC 9027**		***Burkholderia cepacia*** **ATCC 25416**	
**μg/mL**	**Mean**	**SD**	**Mean**	**SD**	**Mean**	**SD**	**Mean**	**SD**
**40**	72%	4%	46%	6%	91%	10%	89%	10%
**20**	71%	5%	6%	10%	75%	16%	87%	12%
**10**	44%	9%	7%	7%	N-Inh	-	42%	12%
**5**	36%	14%	14%	12%	1%	1%	29%	12%
**2.5**	33%	12%	8%	10%	N-Inh	-	25%	14%

N-Inh: No biofilm inhibition recorded at the tested concentration

Table S2. Antitumor activity of Mf-Ag_2ONPs and extract. Mean and standard deviation (SD) are presented. This data was used to generate dose-response curves and calculate the IC_{50} values.

Mf-Ag_2ONPs

**μg/mL**	**HeLa**		**HCT116**		**THJ29T**		**MDA-MB-231**		**NIH3T3**	
	Mean	SD	Mean	SD	Mean	SD	Mean	SD	Mean	SD
**0**	100	0	100	0	100	0	100	0	100	0
**0.39**	98.283		98.3		82.835				82.777	
**0.78**	85.274	11.502	99.847	1.078	85.905	9.274	88.299	1.255	84.546	12.714
**1.56**	78.758	9.156	95.749	7.447	84.855	8.247	79.928	1.022	78.625	10.303
**3.13**	78.922	10.78	81.853	2.713	79.942	3.426	66.741	25.375	70.548	19.279
**3.91**			81							
**6.25**	19.536	2.876	23.42	1.421	51.863	19.93	47.886	13.74	35.26	4.43
**7.81**			32							
**12.5**	19.195	3.578	26.013	8.079	33.039	12.38	41.089	2.168	30.214	3.509
**15.63**			34							

**25**	20.188	3.547	23.833	2.157	30.199	6.996	39.197	4.56	32.922	4.424
**31.25**			36							
**50**	18.727	3.121	24.566	2.253	31.203	5.846	39.066	9.974	37.722	7.195
**100**	22.623		27.609		36.757					

Mf-extract

**mg/mL**	**HeLa**		**HCT116**		**THJ29T**		**MDA-MB-231**		**NIH3T3**	
	Mean	SD	Mean	SD	Mean	SD	Mean	SD	Mean	SD
**0**	100	0	100	0	100	0	100	0	100	0
**0.08**	89.825									
**0.16**	77.095	10.113	99.393	3.371	93.844	6.067	91.508	2.63	83.073	11.92
**0.31**	71.002	9.601	92.512	5.95	88.435	4.21	83.417	1.797	76.762	7.982
**0.63**	69.65	9.382	94.299	7.793	93.465	11.482	83.774	5.928	75.02	8.869
**1.25**	70.629	10.733	87.107	1.16	88.818	8.813	81.346	7.464	72.416	6.034
**2.5**	70.179	8.272	86.016	8.183	80.543	4.184	74.949	11.981	66.782	7.741
**5**	48.497	15.416	58.918	15.442	63.728	13.394	65.761	13.224	62.87	11.015
**10**	18.925	2.278	25.89	7.562	27.134	4.875	41.835	3.696	43.431	7.607
